# An integrated transcriptomic analysis of autism spectrum disorder

**DOI:** 10.1038/s41598-019-48160-x

**Published:** 2019-08-14

**Authors:** Yi He, Yuan Zhou, Wei Ma, Juan Wang

**Affiliations:** 1 Department of Biomedical Informatics, School of Basic Medical Sciences, Peking University, Beijing, 100191 China; 2 Autism Research Center of Peking University Health Science Center, Beijing, 100191 China; 3 The Sixth Medical Center, Chinese PLA General Hospital, Beijing, 100048 China

**Keywords:** Autism spectrum disorders, Bioinformatics

## Abstract

Autism spectrum disorder (ASD) is not a single disease but a set of disorders. To find clues of ASD pathogenesis in transcriptomic data, we performed an integrated transcriptomic analysis of ASD. After screening based on several standards in Gene Expression Omnibus (GEO) database, we obtained 11 series of transcriptomic data of different human tissues of ASD patients and healthy controls. Multidimensional scaling analysis revealed that datasets from the same tissue had bigger similarity than from different tissues. Functional enrichment analysis demonstrated that differential expressed genes were significantly enriched in inflammation/immune response, mitochondrion-related function and oxidative phosphorylation. Interestingly, genes enriched in inflammation/immune response were up-regulated in the brain tissues and down-regulated in the blood. In addition, drug prediction provided several compounds which might reverse gene expression profiles of ASD patients. And we also replicated the methods and criteria of transcriptomic analysis with datasets of ASD animal models and healthy controls, the results from animal models consolidated the results of transcriptomic analysis of ASD human tissues. In general, the results of our study may provide researchers a new sight of understanding the etiology of ASD and clinicians the possibilities of developing medical therapies.

## Introduction

Autism spectrum disorder (ASD) represents a set of neurodevelopmental disorders characterized by two core symptoms, impaired social interaction and restrictive and repetitive behaviors. It is sex-biased that ASD affects boys four to five times more than girls^1^. The pathogenesis of ASD still remains perplexing. Biological researches supported that it was a set of disorders with multiple non-genetic and genetic factors as well as their interactions rather than a single disease^2–6^. About 10–20% of ASD patients have a definite genetic risk^7^. However, the genetic etiology of ASD is heterogeneous. Up till now, hundreds of genes have been found associated with ASD^6^. For example, synaptic genes such as neuroligin 3(*NLGN3*), neuroligin 4X-linked(*NLGN4X*)^8^, SH3 and multiple ankyrin repeat domains 3(*SHANK3*)^9,10^ were certified as the ASD genes. Monogenetic diseases like Fragile-X syndrome and Rett Syndrome associated with the mutations of fragile X mental retardation 1(*FMR1*)/methyl-CpG binding protein 2(*MECP2*) were also found with autistic symptoms^11^. Ubiquitin protein ligase E3A (*UBE3A*) gene coding for E3 ubiquitin-protein ligase was linked to both ASD and Angelman Syndrome^2^. The results above came from the researches of DNA sequence, but they cannot identify the cause of ASD in a large number of cases^11^.

Transcriptomic analysis plays an important role in exploring genome structure and function and identifying genetic networks in aspect of gene transcription. Gene expression patterns in autistic population have been demonstrated in different tissues including lymphoblastoid cell lines, peripheral blood, brain cells and pluripotent stem cell-derived neurons^6,12–14^. Several researches pointed out that aberrant gene expression in the blood of children with ASD involved in transcriptional regulation, biosynthesis of protein, processing of ribosomal RNA and neural-related pathways^13,15,16^. Transcriptome analysis between autistic brain and normal brain identified discrete modules by gene co-expression network analysis: a neuronal module and a module enriched for immune genes and glial markers^12^. Another finding of brain transcriptional association with ASD revealed two abnormal areas of metabolism: mitochondrial oxidative phosphorylation and protein translation^17^.

Gene Expression Omnibus (GEO, https://www.ncbi.nlm.nih.gov/geo/) has collected the raw and processed data for studies of high-throughput gene expression and genomics^18^. To date, GEO has gene expression datasets coming from hundreds of studies related to ASD. Although widely assumed, it remains unknown whether the transcriptomic signatures were consistent among ASD population. Evidence certainly denoted that differential expressed genes in the blood could be an indicator of ASD, however, whether the transcriptomic signatures in different tissues were consistent with each other hasn’t been explored. Whether we could find some potential drugs based on transcriptomic signatures of ASD. The purpose of our study is to systematically explore the status of gene expression between ASD and healthy control by integrating several human datasets. In this work, we chose the transcriptomic datasets from GEO based on several criteria and screened the differential expressed genes by a computational method and carried on a series of bioinformatic analysis. And we attempted to predict potential drugs based on these differential expressed genes. In addition, we also replicated the strategy and criteria of the analysis in transcriptomic datasets of animal model to validate the results of human tissues.

## Results

### Datasets derived from same tissue had bigger similarity

**Figure 1:**
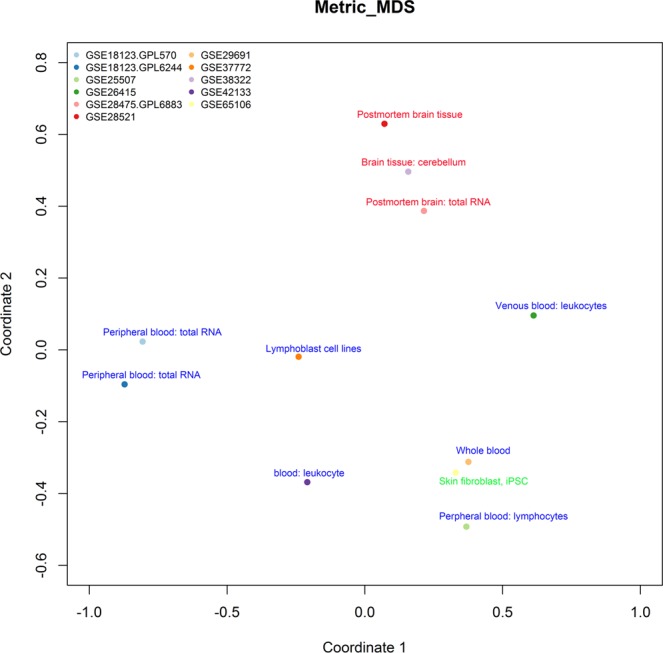
Multidimensional scaling map of 11 series of datasets. Multidimensional scaling analysis was performed among these datasets based on the distance matrix. Each dot represented a series of dataset. For each dataset, the tissue source was noted on the dot, brain tissues were presented by red, blood was presented by blue. Spatial distance among datasets derived from same tissue was smaller than that derived from different tissues (*P* = 0.0013). That meant datasets coming from same tissue had bigger similarity.

To obtain the spatial or geometric representations of the datasets, multidimensional scaling (MDS) was utilized in our study. As shown in Fig. 1, three series of datasets (GSE28475, GSE28521, GSE38322) were derived from brain tissues gathered together, and they were apart from other datasets spatially. In other words, the dimensional distances between any two of three datasets derived from brain tissue were smaller than that between any of them and dataset which came from blood. Similarly, datasets from blood (GSE18123, GSE25507, GSE29691, GSE37772, GSE42133) gathered together. Given the above, datasets derived from the same tissue had the bigger similarity than from different tissues. (The average distance of datasets derived from brain tissues = 1.18, the average distance of datasets derived from blood = 1.40, *P* = 0.0013).

### Gene expression level between brain tissues and blood

**Figure 2:**
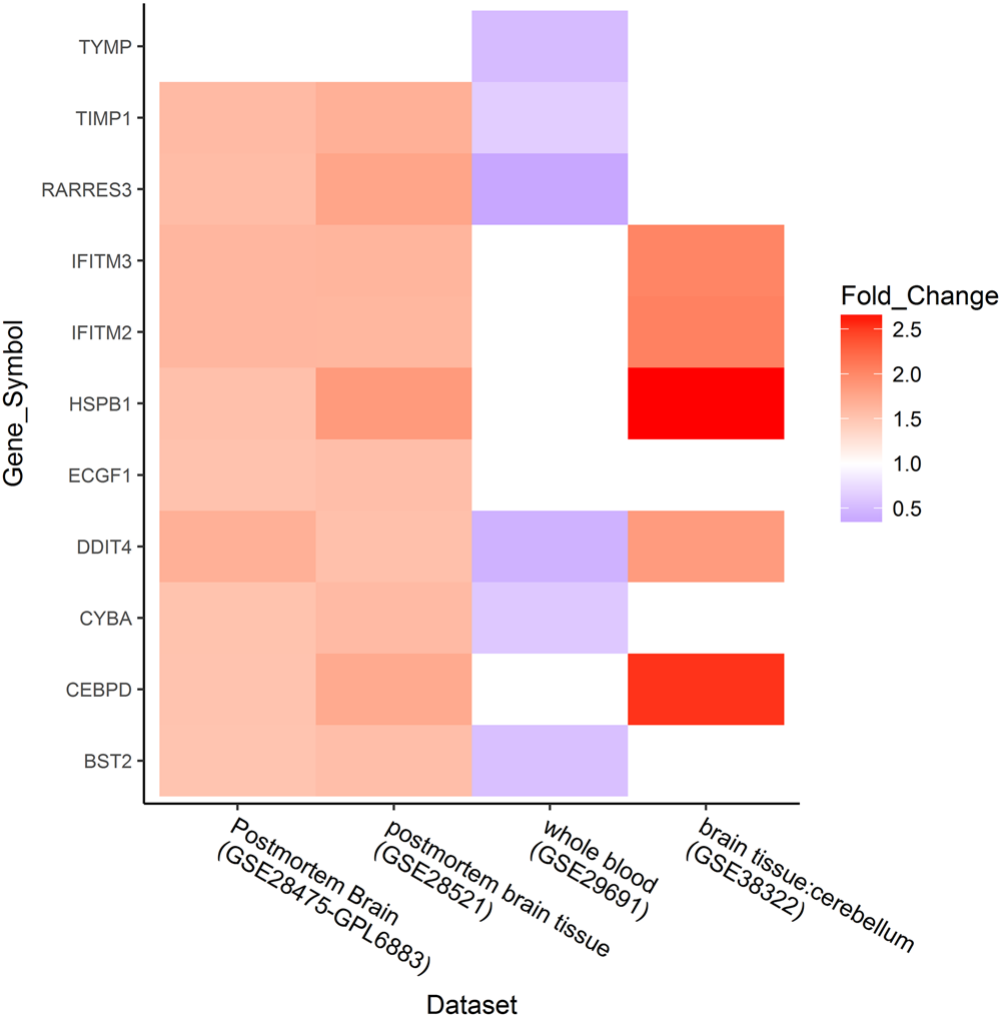
Gene expression levels in different tissues. Each dataset had a differential expressed gene list, gene symbols of y-coordinate were present in at least three datasets. Up-regulated genes were represented by red, and down-regulated genes were blue. The shade of the color reflected the value of fold change of each gene.

Based on the fold change of expression level, we found that differential expressed genes of ASD patients/controls in brain tissues and blood, had different gene expression patterns. The number of up-regulated genes in brain tissues were apparently more than that in blood, meanwhile the number of down-regulated genes in brain tissues was less than that in blood (Supplementary Fig. S1). Especially, several genes up-regulated in brain tissue were down-regulated in blood, like TIMP metallopeptidase inhibitor 1(*TIMP1*), phospholipase A and acyltransferase 4(*RARRES3*), DNA damage inducible transcript 4(*DDIT4*), cytochrome b-245 alpha chain (*CYBA*), bone marrow stromal cell *antigen 2*(*BST2*) (Fig. 2). Namely, there existed a transcriptomic difference in ASD patients’ tissues between brain tissues and blood.

### Functional enrichment of the differential expressed genes

**Figure 3:**
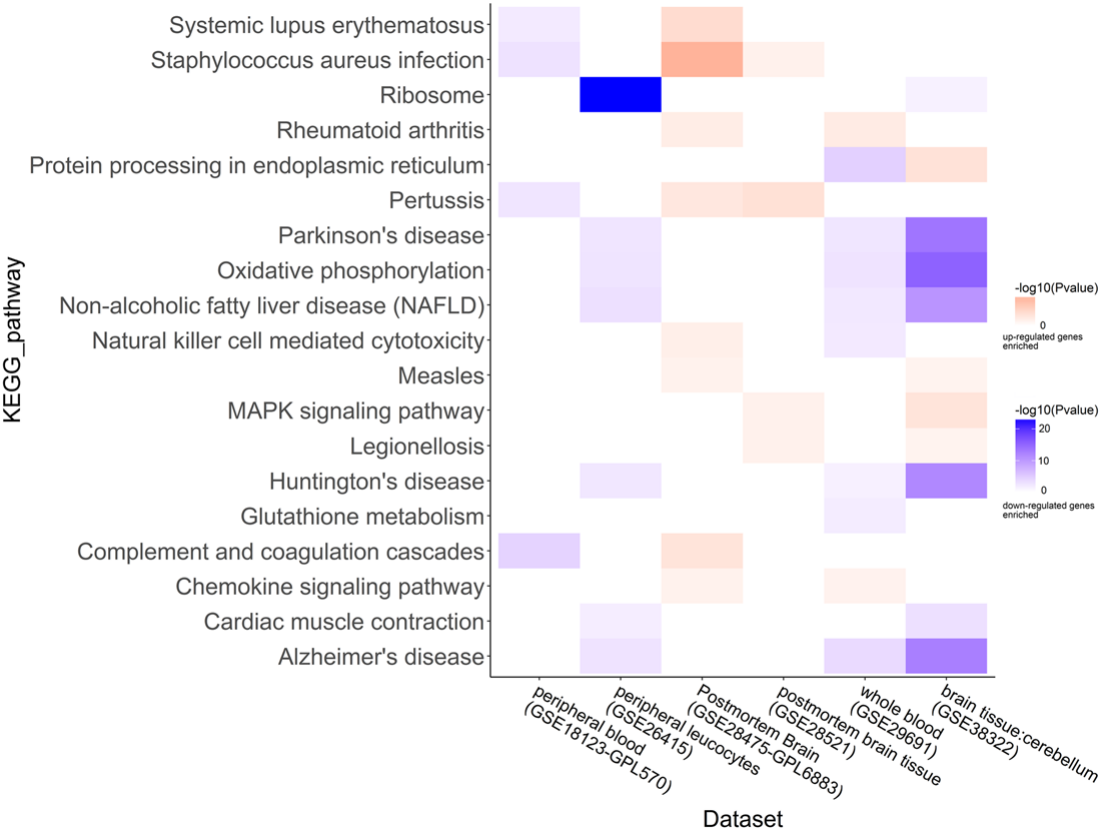
Heat-map for the enriched KEGG pathways. Functional enrichment analysis were performed by DAVID. *P* value of each KEGG pathways was <0.05. KEGG pathways of y-coordinate were the pathways which overlapped in two datasets or more. Up-regulated genes enriched KEGG pathways were represented by red, and down-regulated genes enriched KEGG pathways were blue. The shades of the colors reflected the −log (*P* value) of the enrichment analysis.

**Figure 4:**
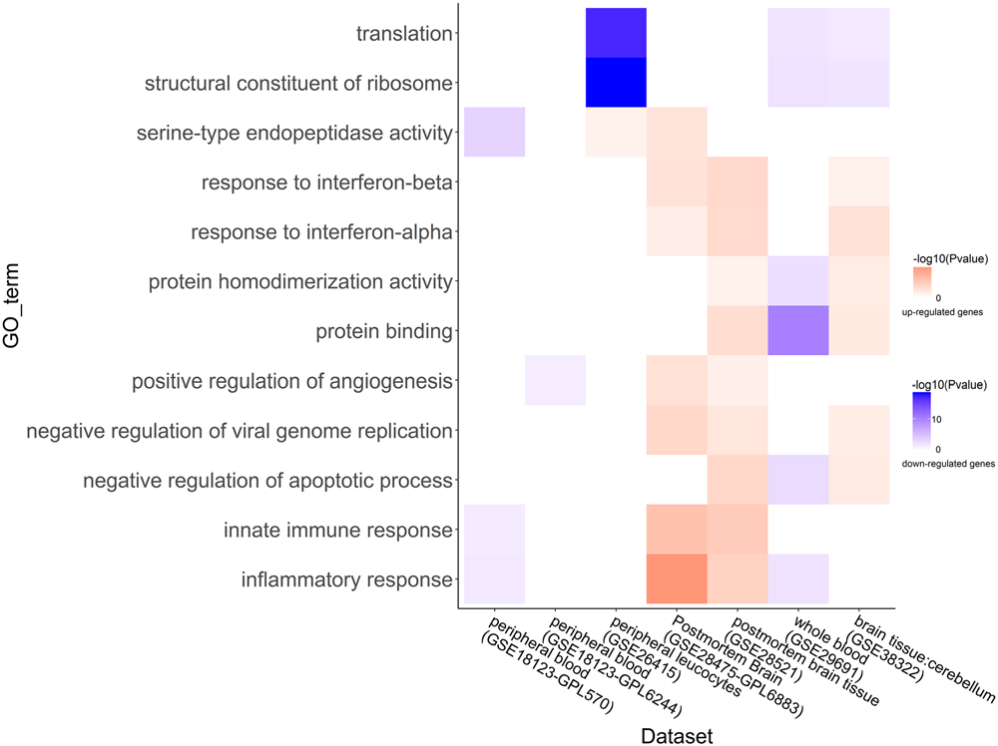
Heat-map for the enriched GO terms. Functional enrichment analysis were performed by DAVID. *P* value of each GO term was <0.05. GO terms of y-coordinate were the pathways which overlapped in three datasets or more. Up-regulated genes enriched GO terms were represented by red, and down-regulated genes enriched GO terms were blue. The shades of the colors reflected the −log (*P* value) of the enrichment analysis.

In terms of fold change and *P*-value of Wilcoxon rank-sum test, each dataset had two gene lists, one for up-regulated genes and the other for down-regulated genes. We input them into the DAVID respectively, and obtained two sets of functional annotations in each dataset. Pathway analysis indicated that multiple pathways associated with inflammation/immune response, mitochondrion-related function and other significantly meaningful pathways (Figs 3 and 4, Supplementary Fig. S2), which were consistent with previous studies^12,17,19,20^. In this work, genes enriched in the KEGG pathway of inflammation/immune related disease, e.g. systemic lupus erythematosus (KEGG pathway identifier: hsa05322), staphylococcus aureus infection (KEGG pathway identifier: hsa05150), etc., were up-regulated in the brain tissues and down-regulated in the blood (Fig. 3). It was similar with the Gene Ontology (GO) terms. Genes enriched in inflammation/immune response were up-regulated in the brain tissues and down-regulated in blood (Fig. 4). Increasing evidence indicated that neuropsychiatric diseases were associated with brain inflammation, such as ASD, schizophrenia^21^. And the immune dysfunction might be the key point for the genetic and environmental factors to develop ASD^22^. However, we were the first to point out that genes enriched in inflammation/immune response were differential expressed in brain and blood. Moreover, we also found down-regulated genes both in the brain tissues and blood were enriched in some significant pathways, such as oxidative phosphorylation and mitochondrion-related function (Fig. 4, Supplementary Fig. S2). This suggested that there might exist energy metabolism disorders in ASD population. Previous studies have shown that ASD might be associated with mitochondrial disorders. About twenty years ago, researchers reported a boy whose origin of the ASD might be the mitochondrial DNA mutation^23^. A systematic review has reported that the prevalence of the mitochondrial disease in general population of ASD was 5.0%, which was much higher than the prevalence of general population^24^. Functional analysis also demonstrated that differential expressed genes enriched in protein translation and ribosome related functions were down-regulated both in brain tissues and blood. Previous study has confirmed that changes in the efficiency of protein translation were related to ASD by using a published set of 1,800 autism quartets and genome-wide variants^25^.

### Drug prediction based on the transcriptomic signatures

The connectivity map (CMap) provides a way to find inverse relationship between disease signature and compound-based signature. In this context, the compounds could potentially present an opportunity to reverse the status of differential expressed genes in ASD population. On account of technical limitation of the online tool, we conducted CMap queries in 5 datasets. And we chose the compounds whose connectivity score ranged from −80 to −100. One dataset (GSE18123-GPL570) was excluded to conduct subsequent analysis because it only had one compound meeting the condition. The compounds overlapping in four datasets were shown in Table 1. Whether these compounds could improve the symptoms of ASD needs to be further explored.

**Table 1:** Compounds overlapping in four datasets.

Compounds	Description
efavirenz	HIV protease inhibitor
SB-525334	TGF beta receptor inhibitor
RHO-kinase-inhibitor-III[rockout]	Rho associated kinase inhibitor
rilmenidine	Adrenergic receptor agonist
PKCbeta-inhibitor	PKC inhibitor
deferiprone	Chelating agent
nifedipine	Calcium channel blocker
cholic-acid	Bile acid

To assess the targets and pathways of the compounds obtained from CMap, we chose the compounds overlapping in more than three datasets as the list of interested drugs derived from human tissues, then used the DrugPattern to conduct enrichment analysis of these drugs. DrugPattern is an online tool for drug set enrichment analysis and contains 7019 drug sets, including indications, adverse reactions, targets, pathways, etc.^26^. The results of DrugPattern were detailed in Supplementary Table S1. The significant drug targets that our interested drugs enriched were 30S ribosome protein, gamma-aminobutyric acid receptor, sodium channel protein, voltage-dependent calcium channel and etc.

### Transcriptomic analysis of ASD animal models validated the results of human tissues

**Figure 5:**
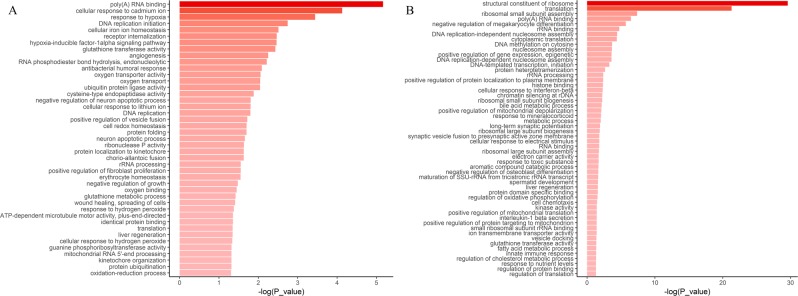
Functional enrichment analysis of differential expressed genes derived from animal models. Functional enrichment analysis were separately performed in differential expressed genes derived from rats and mice. *P* value of each GO term was < 0.05. (**A**) Rattus norvegicus, (**B**) Mus musculus.

After screening in GEO database, we obtained 8 datasets of ASD animal models and healthy controls (Supplementary Table S2). One dataset was from rattus norvegicus, and other seven datasets were from mus musculus. All these samples in the datasets were derived from brain tissues. We defined differential expressed genes and performed functional enrichment analysis and made drug prediction by using the similar strategy and criteria which were used in transcriptomic analysis of human tissues. Our results demonstrated all differential expressed genes derived from animal models were up-regulated (fold change >1.5, *P* value < 0.05, Supplementary Table S2), and none of differential expressed genes was down-regulated. And we separately performed enrichment analysis for differential expressed genes of mus musculus and rattus norvegicus. One dataset (GSE77972) had only one differential expressed gene, so we performed functional enrichment analysis in other 7 datasets and chose statistically significant GO terms. Functional enrichment analysis demonstrated that genes were enriched in oxidative stress related pathway, inflammation/immune response, ribosome and protein translation (Fig. 5), which were consistent with the results of human tissues.

**Figure 6:**
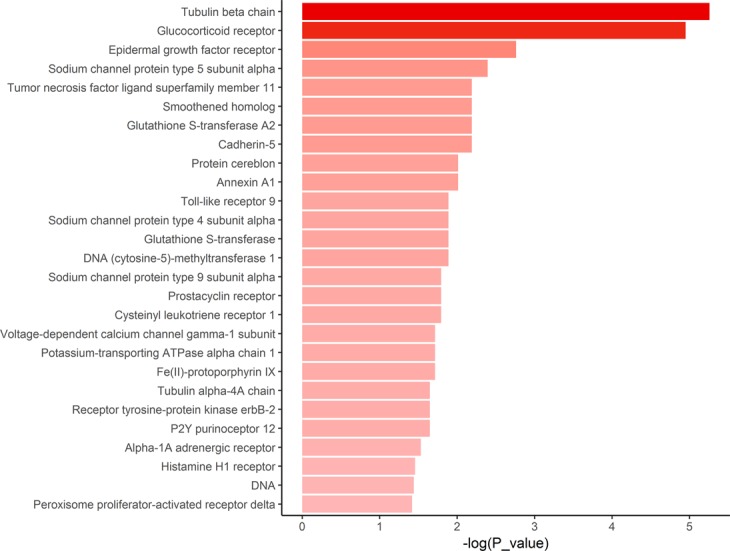
Same drug targets of interested drugs derived from animal models. The compounds overlapping in more than four datasets were defined as the list of interested drugs derived from animal models. Then they were inputted into DrugPattern to perform drug set enrichment. The shade of the color reflected the value of −log(*P* value) of each term.

On account of technical limitation of the online tool, we conducted CMap queries in 6 datasets derived from animal models. After CMap query, the compounds whose connectivity score ranged from −80 to −100 overlapping in more than four datasets were defined as the list of interested drugs derived from animal models. There were 18 drugs overlapping in interested drug lists of human tissues and animal models (Supplementary Table S3). Finally, we performed enrichment analysis of interested drugs of animal models by DrugPattern. The results also suggested that there were several significant drug targets overlapping with the results of human tissues, namely epidermal growth factor receptor, sodium channel protein type5 and type9 subunit alpha, tubulin alpha-4A chain (Fig. 6, Supplementary Table S3).

To validate whether the similar results between human tissues and animal models were generated by random or on account of autism spectrum disorder, we separately performed ten thousand random experiments for enrichment analysis and drug prediction. Random experiment of enrichment analysis and drug prediction based on genes were similar to that based on functional annotations and drugs. We calculated the number of same GO terms derived from randomly enrichment analysis for human tissues and animal models based on the same criteria in our study in each random experiment. Random experiments suggested that the number of same GO terms was mostly zero, which was statistically different from our result (*P* < 0.0001). According to the total number of drugs in CMap database, each random experiment accounted the number of same drugs derived from randomly drug prediction for human tissues and animal models based on the same criteria in our study. Finally, our result that there were 18 drugs overlapping in interested drug lists of human tissues and animal models was statistically different from ten thousand random experiments (*P* < 0.0001) (Supplementary Fig. S3). In general, the results from animal models statistically validated the results of transcriptomic analysis of human tissues.

## Discussion

As a complex disorder which has significant individual differences, a majority of researchers pay more attention to ASD. Mutations in a limited number of genes could not account for a large number of cases in ASD population. Changes in the shape of the entire gene expression distribution suggest alterations at global levels of gene expression regulation. Here we obtained 11 series of datasets from GEO database to explore the gene expression distribution derived from different human tissues. In the previous studies, differential expressed genes were defined according to different fold change. We screened the differential expressed genes based on the same criterion among datasets included in our study. We performed multidimensional scaling analysis among them and concluded that datasets of the same tissue had the bigger similarity compared with the datasets of different tissues. Subsequently, we reinforced the different role of transcriptional regulation between brain tissues and blood by demonstrating with enrichment analysis that, genes enriched in some significant pathways, like inflammation/immune response, were up-regulated in the brain tissues and down-regulated in blood. And we exploited two online tools, CMap and DrugPattern, to find several compounds which may reverse the expression pattern of ASD and explore the possible therapy of ASD. And we replicated the methods and criteria of transcriptomic analysis with animal models and healthy controls, the results were similar to that of human tissues.

Alterations of the transcriptomic regulation could have tissue specificity. For instance, in our study, gene expression patterns in the brain and blood were apparently different. The number of up-regulated genes in the brain tissues was significantly higher than that in the blood. Multidimensional scaling analysis demonstrated datasets derived from brain tissues gathered together spatially. However, the postmortem brain tissues of ASD are scarce, the brain samples of three datasets were procured from the Harvard Brain Tissue Resource Center (www.brainbank.mclean.org). If there were any sample shared by different studies, it would contribute to the dataset similarity. In addition, animal experiment demonstrated that gene expression level might alter along with the fixation times and storage conditions^27^. Blood samples were usually processed fresh, however, human brain tissues were obtained post-mortem. Since lack of the information of sample fixation in GEO database, whether the differences between brain and blood could be at least partially explained by tissue processing need to be further researched.

Immune dysregulation or inflammation, oxidative stress, mitochondrial dysfunction and environmental toxicant exposures are the four major areas implicated in ASD and other psychiatric disorders^28^. A large percentage of publications implicated an association between ASD and these four major areas^28^. Transcriptomic profiling may be particularly important in understanding the pathogenesis of diseases such as ASD where multiple systems are involved. Thus, it is possible that changes of transcriptomic regulation could offer a unifying understanding of multi-systemic effects of ASD. Our results were consistent with previous studies. Differential expressed genes were enriched mainly in inflammation/immune response, mitochondrion-related functions and oxidative phosphorylation. Interestingly, genes enriched in inflammation/immune response were up-regulated in the brain tissues and down-regulated in the blood. ASD children are vulnerable to environmental factors, such as infection, stress or other toxicants exposure^6^. Corticotropin-releasing hormone (CRH) is secreted under stress, and it can stimulate mast cells and microglia along with neurotensin, which results in brain inflammation and neurotoxicity^29^. Mast cells and microglia were found to be activated in brains of children with ASD^30,31^. Increasing evidence indicated that mast cells activation was related to the disruption of blood-brain barrier^29,32,33^. So, inflammation related cytokines may enter into blood across the blood-brain barrier. In fact, alterations in immune function have been reported in ASD patients, such as increased pro-inflammatory cytokine profiles in the cerebrospinal fluid and blood, elevated brain-specific auto-antibodies^34^. Thus, we speculated that when inflammatory cytokines entered the blood, it would inhibit the related-genes expression, which may provide an explanation why genes enriched in inflammation/immune response were up-regulated in brain tissues but down-regulated in the blood.

Risperidone and aripiprazole are the only two drugs which have been approved by the US Food and Drug Administration for the treatment of ASD, however, these two drugs both target irritability rather than social deficits and repetitive behavior^35^. In this work, we described eight overlapping compounds which have the reverse expression profiles of ASD patients, such as efavirenz, rilmenidine, PKCbeta-inhibitor, deferiprone, nifedipine and etc. Extended-release guanfacine, alpha2 agonists, has an effect on hyperactivity, impulsiveness and distractibility in children with ASD^36^. Rilmenidine, a alpha2A-adrenoceptors agonist, has an effect on cardiovascular system in D79N alpha2A-adrenoceptor transgenic mice^37^. Whether rilmenidine could improve some symptoms in children with ASD, like guanfacine, needs to be further explored. Moreover, other compounds also need to conduct some experiments to testify their clinical effects on ASD. In addition, DrugPattern provides us some important information based on the compounds overlapping in more than three datasets. These compounds targeted 30S ribosome protein, gamma-aminobutyric acid (GABA) receptor, sodium channel protein, voltage-dependent calcium channel and etc., which might be potential therapies of ASD. In fact, previous study has verified that mutations in GABA_A_ receptor subunit was associated with epilepsy, autism and other neuropsychiatric disorders^38^. Taken together, our results provided some information to explore the etiology and therapy of ASD.

Simultaneously, our study had some limitations. First, we didn’t conduct false discovery rate (FDR) correction for multiple testing, because the number of differential expressed genes we obtained after FDR correction was too small to perform analysis. Thus we screened the differential expressed genes based on fold change and *P* value of Wilcoxon rank-sum test. Second, we can hardly perform further experimental study to explore the compounds found in our study and ASD on account of limited resources. Because of the complexity of ASD, it is important to take advantage of the bioinformatics methods to explore the pathogenesis and therapy of this disorder.

## Methods and Materials

### Transcriptomic datasets of ASD

We searched the transcriptomic datasets from GEO database based on the key words “autism”, “autism spectrum disorder” and “ASD”, then picked up the datasets by using the following criteria: (1) samples derived from human tissues, (2) samples in each dataset must include ASD and healthy controls, (3) gene expression datasets which are tested by DNA microarray must be on one channel. Finally, we acquired 11 series of transcriptomic datasets (Table 2).

**Table 2:** Datasets from GEO database.

GEO accession number	Platform	Tissue	ASD:healthy control
GSE18123^20^	GPL570	peripheral blood	104:82
GSE18123^20^	GPL6244	peripheral blood	66:33
GSE25507^13^	GPL570	peripheral lymphocytes	82:64
GSE26415^15^	GPL6480	peripheral leucocytes	21:42
GSE28475^46^	GPL6883	postmortem brain tissues	52:71
GSE28521^12^	GPL6883	postmortem brain tissues	39:40
GSE29691	GPL570	lymphoblastoid cell lines (LCLs)	2:13
GSE37772^16^	GPL6883	lymphoblast cell lines	233:206
GSE38322^17^	GPL10558	postmortem brain tissues	18:18
GSE42133^19,47^	GPL10558	peripheral leucocytes	91:56
GSE65106^14^	GPL6244	skin fibroblast, iPSC, iPSC-derived neural progenitors, and iPSC-derived neurons	21:38

The concept of differential expressed genes is the genes showing different expression level in human tissues between ASD patients and healthy controls. We used R software to perform our analysis. Specially, each gene’s differential expression level can be evaluated by fold change and Wilcoxon rank-sum test. If we defined differential expressed genes according to FDR, the number of differential expressed genes was too small to perform the sequent analysis (Supplementary Table S4), so we chose the genes meeting the conditions that fold change >1.5 or <1/1.5 with *P* value < 0.05 as the differential expression genes. Calculated differential expressed genes in different datasets have been summarized in Supplementary Table S5.

### Multidimensional scaling analysis of datasets

MDS is a method of quantitatively estimating the similarity among groups of items, and it refers to a set of statistical techniques that are used to reduce the dimensions of the data, so as to find out the visual appreciation of the underlying relational structures^39^. The result of MDS is a “map” which spatially denotes the relationships among items, wherein similar items are located proximal to one another, and dissimilar items are located proportionately further apart^39^. Given that, we performed MDS to quantify similarity judgements among datasets. First, we separately calculated fold change of all genes for each dataset. Then we carried out the spearman rank correlation analysis between each two datasets based on fold change. And we obtained the Euclidean distance between each two datasets according to correlation coefficients. Finally we chose 2 as the value of dimension and used distance matrix of these datasets to perform MDS by R3.4.4 software stats package.

### Enrichment analysis of differential expressed genes

The enrichment analysis of interesting gene lists which are derived from microarray or next-generation RNA sequencing (RNA-seq) is a basic method to find significant information about the biological pathways^40^. Compared with the analysis of all differential expressed genes together, enrichment analysis of up- and down-regulated genes separately was proven to be more powerful^41^. For each dataset in our study, we conducted enrichment analysis of up- and down-regulated gene lists respectively by the DAVID v6.8 (https://david.ncifcrf.gov/)^40,42^.

### Drug prediction

The CMap database contains over 7000 expression profiles tested in five human cell lines, and utilizes gene expression profiling to reveal the associations among genes, chemicals and biological conditions such as disease^43,44^. The next generation connectivity map termed as L1000 is more than a 1000-fold scale-up of the CMap (https://clue.io/L1000)^45^. The set of differential expressed genes in our study can be used to query and compare against a large reference catalogue of gene expression profiles derived from drug or other perturbagen treatment cell lines in the connectivity map. The CMap provides an online tool to perform CMap queries against the chemical reference catalogues. For the query CMap, up-regulated genes are essential, however, down-regulated genes are optional. The number of valid genes inputted into the query CMap should be limited to 10–150. So we chose the datasets whose number of valid up-regulated genes exceeded 10. If the valid up-regulated genes exceeded 150, we chose the top 150 valid genes according to fold change to conduct CMap query. Finally, we acquired 5 datasets meeting the conditions to perform CMap query (GSE18123-GPL570, GSE28475-GPL6883, GSE28521, GSE29691, GSE38322). The connectivity score ranges from +100 to −100. A positive connectivity score represents a positive correlation and a negative connectivity score denotes a negative correlation between our differential expressed gene list and a reference profile derived from an individual chemical perturbation. Thus a negative score may imply that exposure to a specific chemical may reverse the expression pattern of ASD. We chose the compounds whose connectivity score were between −80 to −100 in each dataset. The compounds overlapping in more than three datasets were inputted into DrugPattern (http://www.cuilab.cn/drugpattern) in order to mine regular rules and patterns behind a list of drugs^26^.

### Transcriptomic analysis of animal model

Increasing number of datasets will help to better understand the gene expression profiles of ASD, so we also analyzed transcriptomic data derived from ASD animal models and healthy controls. Similarly, we screened gene expression datasets from the GEO database according to the following criteria: (1) the samples are from animals, (2) there are ASD animal models and healthy controls in each dataset, (3) the number of ASD animal models and healthy controls both should be greater than four in each dataset. Finally, we obtained 8 series of datasets (Supplementary Table S2). Then, we identified differential expressed genes between ASD models and healthy controls according to fold change >1.5 or fold change <1/1.5 and *P* value < 0.05, and performed enrichment analysis of these differential expressed genes by using DAVID, and made drug prediction by using CMap and DrugPattern. The number of valid genes inputted into the query CMap should be also limited to 10–150. So we performed CMap query in 6 datasets (GSE34058, GSE62594, GSE63303, GSE77971, GSE99277, GSE117327). Similarly, we chose the compounds whose connectivity score were between −80 to −100 in each dataset. The compounds overlapping in more than four datasets were defined as the list of interested drugs derived from animal models. Then they were inputted into DrugPattern to perform drug set enrichment.

## Supplementary information

Supplementary Materials

Supplementary Table S1

Supplementary Table S2

Supplementary Table S3

Supplementary Table S5

## Data Availability

All data generated or analyzed during this study are included in this published article (and its supplementary information files).
