# Supplementary material for: An integrated transcriptomic analysis of autism spectrum disorder
Source: Sci Rep. 2019 Aug 14;9:11818. doi: 10.1038/s41598-019-48160-x (PMC6694127; doi:10.1038/s41598-019-48160-x)
Supplement: Supplementary file 1 — Supplementary Materials [file 41598_2019_48160_MOESM1_ESM.pdf]

An integrated transcriptomic analysis of autism spectrum disorder

Yi He[1,2], Yuan Zhou[1], Wei Ma[3], Juan Wang[1,2,*]

[1]Department of Biomedical Informatics, School of Basic Medical Sciences, Peking University, Beijing, 100191, China.

[2]Autism research center of Peking University Health Science Center, Beijing, 100191, China.

[3]Central Laboratory, General Navy Hospital of PLA, Beijing, 100048, China.

* Corresponding authors: wjuan@hsc.pku.edu.cn

Supplementary Figures and Tables

Supplementary Figures

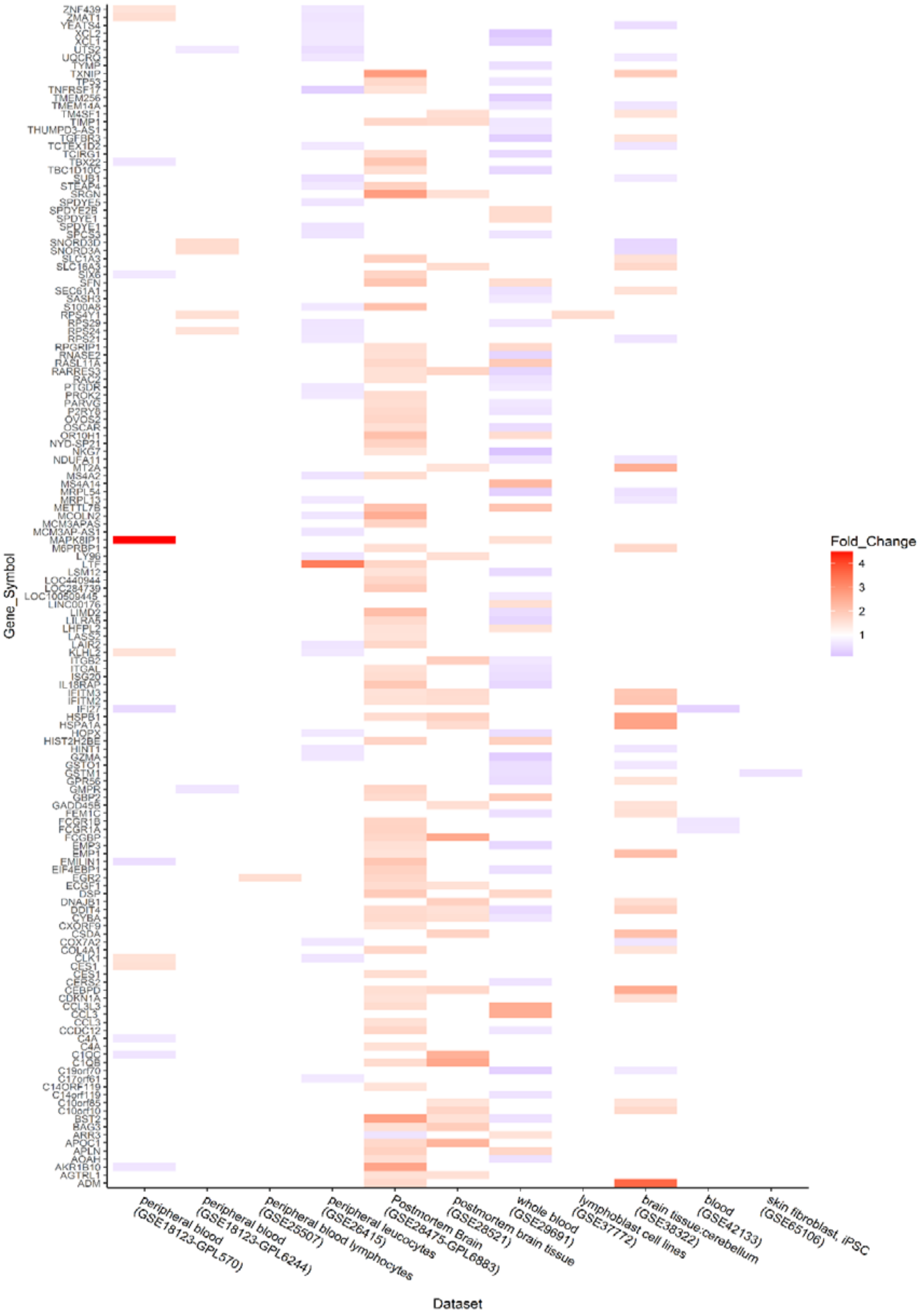

Supplementary Fig.S1: Gene expression levels in different tissues. Each dataset had a differential expressed gene list, gene symbols of y-coordinate were present in at least two datasets. Up-regulated genes were represented by red, and down-regulated genes were blue. The shade of the color reflected the value of fold change of each gene.

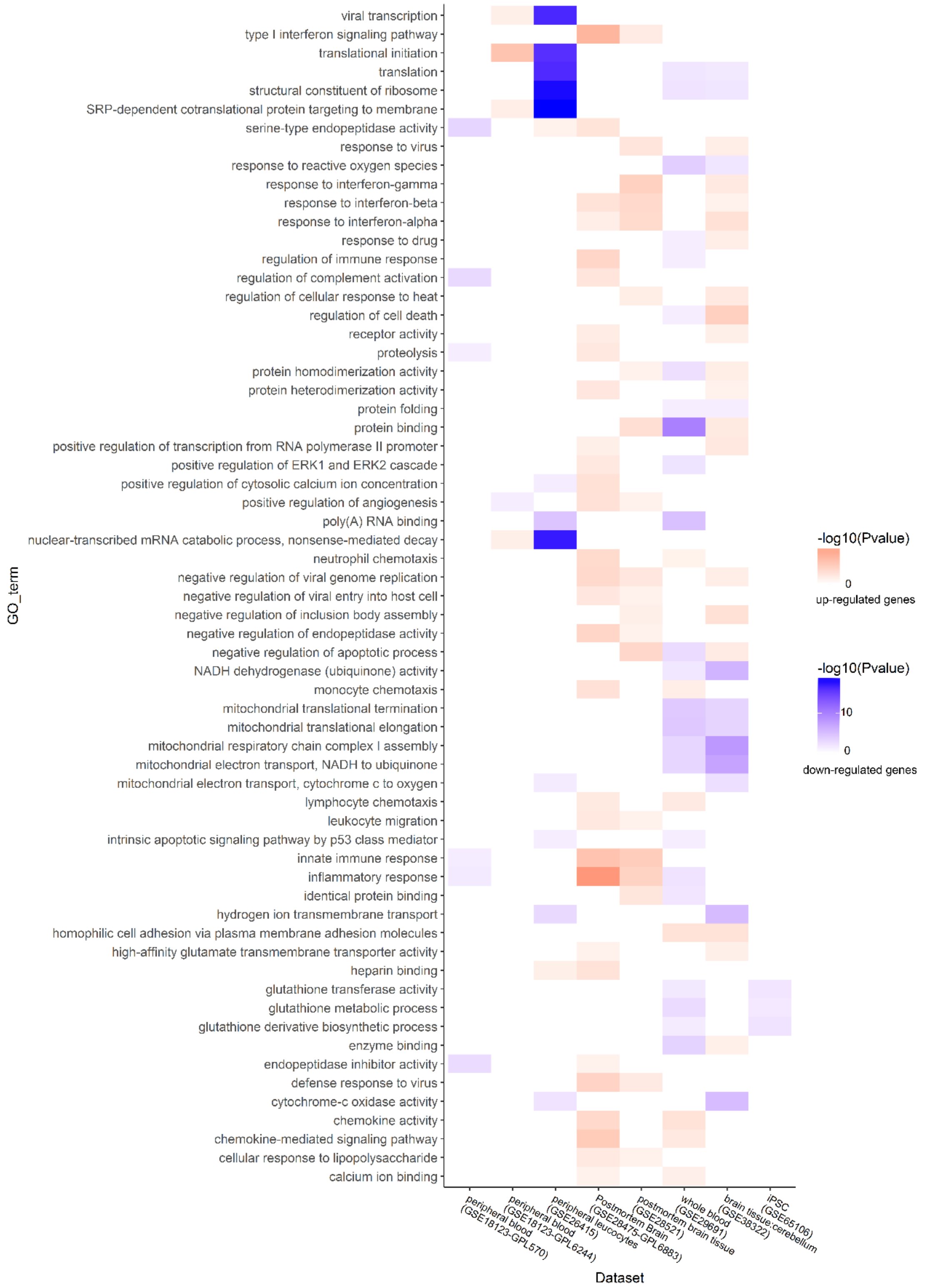

Supplementary Fig.S2: Heat-map for the enriched GO terms. GO terms of functional annotations overlapped in two datasets or more. Up-regulated genes enriched GO terms were represented by red, and down-regulated genes enriched GO terms were blue. The shades of the colors reflected the –log (*P* value) of the enrichment analysis.

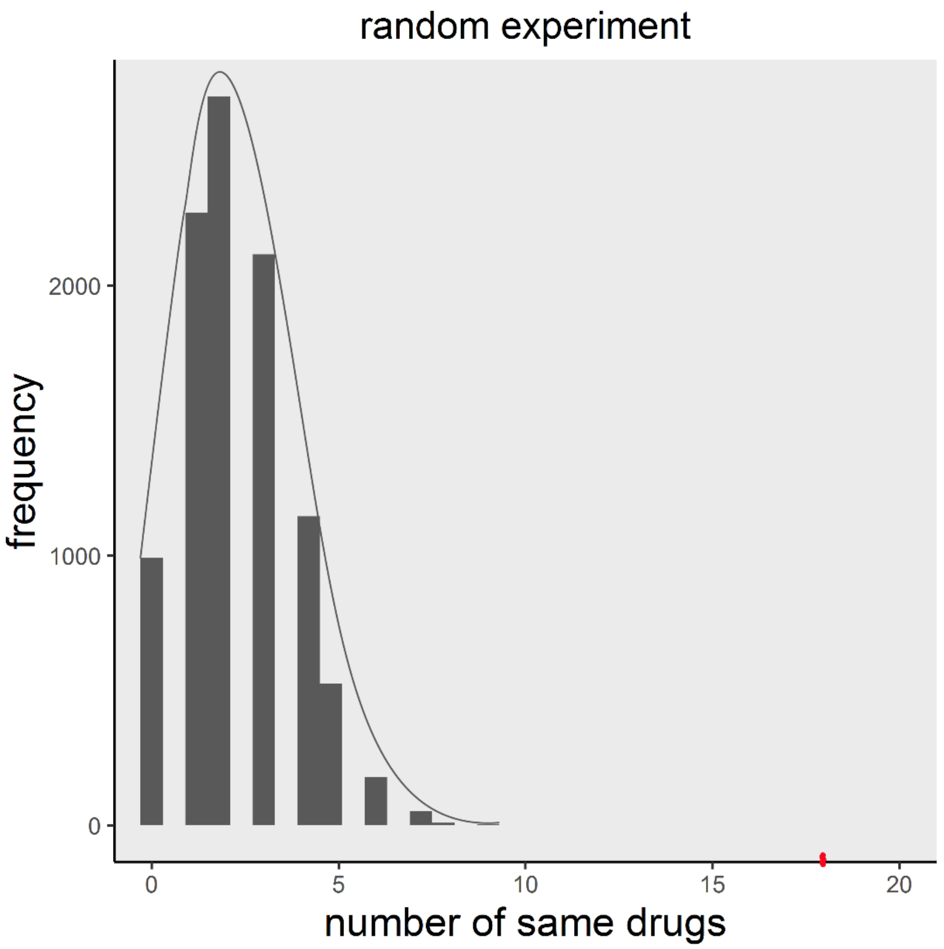

Supplementary Fig.S3: The results of random experiments of drug prediction. Ten thousand random experiments were performed to statistically verify the similar results derived from human tissues and animal models. In each random experiment, according to the criteria of drug prediction in our study, we calculated the number of same drugs between randomly drug prediction for human tissues and animal models. X-coordinate represented the number of same drugs, Y-coordinate represented the frequency of the number of same drugs during the ten thousand random experiments. Red dot represented the actual number of same drugs in our study, $P<0.0001$.

Supplementary Tables

Supplementary Table S1: The results of DrugPattern of interested drug list derived from human tissues. The compounds overlapping in more than three datasets were inputted into DrugPattern, the information of these compounds was described in the table, such as, the similar adverse drug reaction, the same anatomical therapeutic chemical classifications, the same chemical structure classifications, the same disease,

the same pathways and the same target.

Supplementary Table S2: The information about datasets derived from animal models and the calculated differential expressed genes in each dataset. Easy to see, differential expressed genes in each dataset were up-regulated (fold change>1.0, *P* value<0.05).

Supplementary Table S3: The interested drug list derived from animal models and the results of DrugPattern. There are 18 compounds overlapping in the interested drug lists from human tissues and animal models. The results of DrugPattern, like the same pathways and the same target, by using these 18 compounds were described in the table.

Supplementary Table S4: Differential expressed genes selected by FDR<0.01. If we selected differential expressed genes according to FDR<0.01, there were only 7 differential expressed genes so that we couldn't perform the following analysis. That's why we select differential expressed genes according to fold change and *P* value of Wilcoxon rank test.

**Datasets**	**Gene_ID**	**Gene_symbol**	**fold_change**	**P_value**	**FDR**
GSE18123-GPL6244	26851	SNORD3B-1	1.6392956	3.21E-07	0.006794
	780851	SNORD3A	1.6392956	3.21E-07	0.006794
	780852	SNORD3B-2	1.6392956	3.21E-07	0.006794
	780853	SNORD3C	1.6392956	3.21E-07	0.006794
	780854	SNORD3D	1.6392956	3.21E-07	0.006794
GSE28521	4744	NEFH	0.617576	6.64E-07	0.006315
GSE37772	6192	RPS4Y1	1.647246	1.77E-11	3.28E-07

Supplementary Table S5: Differential expressed genes in each dataset derived from human tissues. In each dataset, detailed information about each gene was presented in the table, such as gene id, mean of raw data for ASD patients and healthy controls, fold change and *P* value of Wilcoxon rank sum test.
